# Evaluation of the effects of different sand particles that used in dental implant roughened for osseointegration

**DOI:** 10.1186/s12903-018-0509-3

**Published:** 2018-03-20

**Authors:** Mehmet Emre Yurttutan, Ahmet Keskin

**Affiliations:** Faculty of Dentistry, Department of Oral and Maxillofacial Surgery, Ankara University, Atatürk Mah. Gazi Cad. No:19, Ankara, Turkey

**Keywords:** Dental implant, Osseointegration, Resonance frequency analysis, Removal torque test, Sandblasting

## Abstract

**Background:**

Successful dental implant treatment is directly related to osseointegration. In achieving osseointegration, the surface property of the implant is of great importance. Sandblasting is the most commonly used basic method for modifying the surface. Many companies use different sand particles for surface roughening and claim their sand is the best. This leads clinicians to mix their minds in product selection. In this study, we tried to find the appropriate sand material by working objectively without praising any brand. We believe that the results of the study will help clinicians choose the right dental implant. In this study, machined-surfaced implants and implants sandblasted with Aluminum oxide (Al_2_O_3_), Titanium dioxide (TiO_2_) and Silicon dioxide (SiO_2_) were compared via biomechanical testing.

**Methods:**

For the study, four 2 year-old sheep, weighing 45 kilograms (kg), were used. Eight implants (Al_2_O_3_, TiO_2_, and SiO_2_ sandblasted implants and machined-surfaced implants), each with different surface characteristics, were inserted into the bilateral tibia of each sheep under general anesthesia. Results of the initial Resonance Frequency Analysis (RFA) were recorded just after implant insertion. The sheep were then randomly divided into two groups, each with 2 sheep, to undergo either a 1-month or a 3-month assessment. At the end of the designated evaluation period, RFA and removal torque tests were performed.

**Results:**

Although there were no statistically significant differences between the groups, the implants sandblasted with Al_2_O_3_ showed a higher Implant Stability Quotient (ISQ) and removal torque value at the end of the 1st and 3rd month.

**Conclusions:**

In short, the results of the study demonstrate that Aluminum oxide is superior to other sand particles.

## Background

In recent years, there has been a significant increase in the worldwide usage of dental implants. At present, screws and root-shaped intraosseous implants produced from Titanium aluminium vanadium (TiAIV), an alloy containing 6% aluminum and 4% vanadium, are the most popular [1–3]. The clinical success of dental implants is directly related to the osseointegration that takes place after the healing period [4]. The surface property of dental implants is the leading factor affecting osseointegration, and thereby, the success of the implant [5].

Once an implant is produced by the Computer Numerical Control machine, it is cleaned and polished, which is followed by roughening of the surface via application of one or more of the modification techniques [6]. The most common and basic technique used to roughen the surface of a titanium implant is sandblasting, a process involving the injection of hard ceramic particles onto the implant surface. Sprayed particles create deep cleavages via a cutting effect on the implant surface. Roughening is then performed by plucking small filler particles from the surface [7]. The primary materials used in the sandblasting process are Aluminum oxide (Al_2_O_3_), Titanium dioxide (TiO_2_), Silicon dioxide (SiO_2_), hydroxyapatite powders and silicate glass [8]. Comparisons between polished surface (machine-surfaced) and sandblasted surface implants indicate that there are more bone-to-implant contacts in the latter [9].

The first step in forming the implant surface is sandblasting. Acid etching and subsequent modifications are carried out after the completion of the sandblasting step. It is therefore important that the sandblasting is performed with a suitable sand type. Many companies use different sand particles for surface roughening and claim their sand is the best. This leads clinicians to mix their minds in product selection. In this study, we tried to find the appropriate sand material by working objectively without praising any brand. We believe that the results of the study will help clinicians choose the right dental implant. With that in mind, the aim of this study is to determine the ideal sand particles by comparing the early-term and long-term osseointegration characteristics of implants sand-blasted with Al_2_O_3_, TiO_2_ and SiO_2_ in an experimental sheep model.

## Methods

The study was approved by the Animal Ethics Commission of Ankara University in accordance with the European Union Council Directive. Animals were housed and operated on in the Research and Application Farm of the Veterinary Faculty of Ankara University, and the research was conducted in compliance with the animal research guidelines of the Veterinary Faculty, Ankara University. Four female sheep, at 2 years of age and weighing 45 kilograms (kg), were used in the study. The animals were monitored for 1 week and fed with a standard diet. Before the surgery, all animals fasted for 24 h.

A total of 64 cylindrical implants, 4.0 mm (mm) in diameter and 10 mm in length, were used in the study. All of the implants were produced by our project partner, Genamer Technology. A total of 16 implants, eight right and eight left, were applied to each sheep. The type of implant was randomly applied.

For the study, three different test groups, each with an average roughness, and one control group were created, as shown below:

1. Test group sandblasted with Al_2_O_3_
2. Test group sandblasted with TiO_2_
3. Test group sandblasted with SiO_2_
4. Control group without sandblasting (Machined)

The average particle size of the sand particles was 180-200 μm. According to their group placement, 2 Al_2_O_3_ sandblasted implants, 2 TiO_2_ sandblasted implants, 2 SiO_2_ sandblasted implants, and 2 machined implants (control group) were placed into the proximal tibia of the sheep. The same procedure was also applied to the other tibia, resulting in 12 implants being used in the experimental groups and four implants being used in the control group. The aim here was to have the implants placed in the same animal and the same tibia for both the experimental group and the control group (Table 1). For early-stage assessment (1 month), two of the four animals were used, while for late-stage assessment (3 month), the other two animals were used.

**Table 1:** Types of implants and numbers for each animal

Implant groups sandblasted with	Number of implants used in right tibia	Number of implants used in left tibia	Total
Al_2_O_3_	2	2	4
TiO_2_	2	2	4
SiO_2_	2	2	4
Control group	2	2	4
Total	8	8	16

All surgical procedures were performed under sterile conditions with general anesthesia. First, the surgical area was shaved, washed and disinfected with povidone iodine. To further reduce the risk of postoperative infection, the sheep were treated with antibiotics (Iesefs 1 g, Sefriakson Na intramuscularly [IM] [I.E. Ulagay Ilac, Istanbul, Turkey] before the operation, and 1.5 g IM for 5 days after the operation). Xylazine (Rompun, 0.2- 0.5 ml/kg IM., Bayer, Istanbul, Turkey) and diclofenac potassium (dikloran non-steroid anti-inflammatory IM, 1 mg/kg, Bilim Ilac, Istanbul, Turkey) were administered as premedication. General anesthesia was administered using an intravenous (IV) injection of pentobarbital and maintained by isoflurane 3.5% (volume/volume) (Forane, Abbott Laboratories, Rungis Cedex, France), which was administered through an endotracheal tube.

**Fig. 1:**
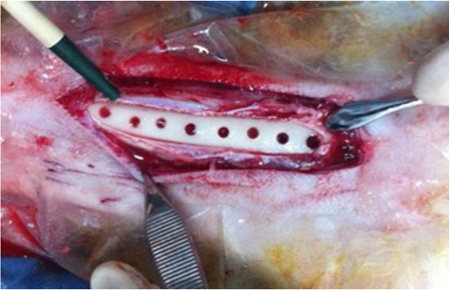
Image of prepared implant cavities

After the animals received general anesthesia, a mid-crestal incision was made in the metaphyseal region of the tibia. After the skin and periosteum was removed, the bone was reached separately. The areas where the implants were to be placed were marked with pointed burs. Then, 10 mm deep implant cavities were prepared using pilot drills with a diameter of 2 mm, followed by a 2.8 mm 2nd drill and lastly, a 3.5 mm final drill. Implants with a diameter of 4.0 mm and a length of 10 mm were placed in the prepared cavities. A total of 16 implants were used (Fig. 1).

**Fig. 2:**
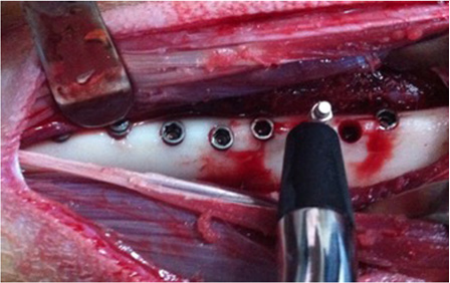
Measurement of stability with Osstell Mentor device

The Osstell Mentor device (Integration Diagnostics AB, Göteborg, Sweden) was used to measure the stability of all implants before closing the flap (Fig. 2). The measurements were performed in five directions (anterior, posterior, right, left and above). The mean value was used to determine the final ISQ of each implant. Afterwards, the fascia, muscle layers and skin were sutured. The operation site was sprayed with oxytetracycline HCl (Terramycin Wound Spray), and the legs were splinted to prevent trauma and tibia fractures. Antibiotic and anti-inflammatory drug use was continued for 5 days. After 2 weeks, the splint and the sutures were removed from the skin. During the course of the study, the animals were regularly provided with food and water. The general health status of all experimental animals was monitored during the healing period, and signs of any infection in the wound areas were carefully checked. At the completion of the healing period, there were no problems detected in any of the animals. One month after the operation, two of the experimental animals were sacrificed, and the implants placed on the tibia were uncovered. The stability of the implants was measured based on the Implant Stability Quotient (ISQ) value, using the Osstell Mentor device. Then, the removal torque test was applied. The same measurements were applied to the other two experimental animals 3 months after they had undergone the operation.

The removal torque test was performed after the ISQ values of the implants were measured. To perform the torque tests, the bone was placed in a vise, and the implant pieces were screwed onto the implant. After the probe of the digital torque meter (Mark-10, MGT 12, USA) was secured onto the implant bearing members, the removal force was applied slowly and incrementally in the counterclockwise direction. The process was completed when the implant began to rotate within the bone cavity. The highest torque value recorded on the digital screen at the time of breakage was measured in the form of Ib-in and recorded in the form of newton centimeter (N/cm).

## Results

### Resonance frequency analysis finding

Table 2 shows the ISQ values of the four main groups constituting the 1-month experimental study. The initial and 1-month ISQ values of the groups were evaluated by the Wilcoxon test.

**Table 2:** Statistical analysis of initial and last ISQ value in 1 month experimental animals

		1 month experimental animals						Wilcoxon test	Kruskal Wallis test
		n	Mean	Min.	Max.	Med.	Standard deviation	p	p
Al_2_O_3_	Initial ISQ	8	56.12	51	60	57	3.22	0.155	0.763
	1-month ISQ	8	58.62	51	68	59	5.39		
TiO_2_	Initial ISQ	8	55.75	48	61	57	4.49	0.664	
	1-month ISQ	8	54.37	45	69	55	7.44		
SiO_2_	Initial ISQ	8	51.62	39	59	54	6.45	0.102	
	1-month ISQ	8	53.87	39	61	55.5	6.79		
Control	Initial ISQ	8	53.5	43	59	55	5.75	0.277	
	1-month ISQ	8	53.62	14	66	57.5	16.91		

Regarding the differences between the 1-month and initial ISQ values according to the separate groups, there was no statistically significant increase between the 1-month ISQ values (mean: 58.62 ± 5.39) and the initial ISQ values (mean: 56.12 ± 3.22) in the implant group sandblasted with Al_2_O_3_ (*p* = 0.155); there was no statistically significant increase between the 1-month ISQ values mean: 54.37 ± 7.44) and the initial ISQ values mean: 55.75 ± 4.49) in the implant group sandblasted with TiO_2_ (*p* = 0.664); there was no statistically significant increase between the 1-month ISQ values mean: 53.87 ± 6.79) and the initial ISQ values mean: 51.62 ± 6.45) in the implant group sandblasted with SiO_2_ (*p* = 0.102); and there was no statistically significant increase between the 1-month ISQ values mean: 53.62 ± 16.91) and the initial ISQ values in the control group (mean: 53.5 ± 5.75) (*p* = 0.277).

In the 1-month experimental group, the increase in the ISQ values from the initial ISQ measurement to the 1-month ISQ of all experimental groups and the control group was assessed by the Kruskal Wallis test, from which it was found that the change in the ISQ values of all groups was statistically insignificant (*p* = 0.763). The 1-month ISQ values of these four groups were compared with each other, and no statistically significant difference was found between the groups (*p* = 0.425).

Table 3 shows the ISQ values of the four groups in the 3-month experimental study. The initial and 3-month ISQ values of the groups were evaluated using the Wilcoxon test, where a statistically significant increase was found between the 3-month ISQ values mean: 62.75 ± 6.43) and the initial ISQ values mean: 55.62 ± 5.65) in the Al_2_O_3_ group (*p* = 0.018); no statistically significant increase between the 3-month ISQ values mean; 57.87 ± 7.18) and the initial ISQ values mean: 58.5 ± 1.77) in the implant group sandblasted with TiO_2_ (*p* = 0.61); and no statistically significant increase between the 3-month ISQ values mean: 59.62 ± 3.37) and the initial ISQ values mean: 58.12 ± 3.44) in the implant group sandblasted with SiO_2_ (*p* = 0.131). In the control group, a statistically significant increase was observed between the 3-month mean: 58.5 ± 5.04) and the initial ISQ values mean: 54.75 ± 5.03) (*p* = 0.007).

**Table 3:** Statistical analysis of initial and 3-month ISQ value

			3 months experimental animals					Wilcoxon test	Kruskal Wallis test
		n	Mean	Min	Max	Med	Standard deviation	p	p
Al_2_O_3_	Initial ISQ	8	55.62	44	62	57	5.65	0.018	0.037
	3-month ISQ	8	62.75	56	74	61.5	6.43		
TiO_2_	Initial ISQ	8	58.5	55	60	59	1.77	0.61	
	3-month ISQ	8	57.87	49	71	57.5	7.18		
SiO_2_	Initial ISQ	8	58.12	55	65	56	3.44	0.131	
	3-month ISQ	8	59.62	53	63	61.5	3.37		
Control	Initial ISQ	8	54.75	45	61	55	5.03	0.007	
	3-month ISQ	8	58.5	48	64	59.5	5.04		

In the 3-month experimental group, the amount of increase from the initial to the 3-month ISQ of all experimental and control groups was assessed using the Kruskal Wallis test, which showed a statistically insignificant change in the ISQ values of all groups (*p* = 0,037). Binary comparisons were made with the Mann Whitney test, which indicated that the highest ISQ increase occurred in the group sandblasted with Al_2_O_3._

### Removal torque test findings

All of the sheep used in the study were subjected to a removal torque test at the end of the follow-up period. Four different implant groups were compared, and the data derived from all groups were evaluated.

In the 1-month experimental group, the differences between the implant groups sandblasted with Al_2_O_3_ (mean: 46.11 ± 17.4 N/cm), sandblasted with TiO_2_ (mean: 37.95 ± 14.1 N/cm), and sandblasted with SiO_2_ (mean: 40.27 ± 20.93 N/cm) and the control group (mean: 42.75 ± 17.03 N/cm) were evaluated using the Kruskal Walls test. According to the results, there were no statistical differences between the torque values of the groups (*p* = 0.656) (Table 4).

**Table 4:** Statistical analysis of 1-month removal torque test data (Newton/cm)

		1 month experimental animals					Kruskal Walls test
	n	Mean	Min.	Max.	Med.	Standard deviation	p
Al_2_O_3_	8	46.11	25.76	80.89	45.5	17.4	0.656
TiO_2_	8	37.95	20.58	65.47	37.52	14.10	
SiO_2_	8	40.27	21.26	81.56	33.92	20.93	
Control	8	42.75	20.93	77.51	40.5	17.03	

In the 3-month experimental group, the differences between the implant groups sandblasted with Al_2_O_3_ (mean: 109.06 ± 33.36 N/cm), sandblasted with TiO_2_ (mean: 93.79 ± 14.03 N/cm), and sandblasted with SiO_2_ (mean: 83.19 ± 21.45 N/cm) and the control group (mean: 82.06 ± 21.79 N/cm) were evaluated using the Kruskal Walls test. The results showed no statistically significant differences between the torque values of the groups (*p* = 0.107) (Table 5).

**Table 5:** Statistical analysis of 3-month removal torque test data (Newton/cm)

		3 month experimental animals					Kruskal Walls test
	n	Mean	Min.	Max.	Med.	Standard deviation	p
Al_2_O_3_	8	109.06	45.67	139.16	124.03	33.36	0.107
TiO_2_	8	93.79	66.15	114.07	94.73	14.03	
SiO_2_	8	83.19	48.15	104.51	89.6	21.45	
Control	8	82.06	59.4	124.53	76.67	21.79	

In cases where the *p* value was close to α, binary comparisons were made with the Mann Whitney test. In the statistical binary comparisons performed on the experimental animals in the 3rd month, the highest torque value was achieved by the implant group sandblasted with Al_2_O_3_, followed by the groups sandblasted with TiO_2_ and SiO_2_.

In the study, the 1st and 3rd month torque values of all groups were compared using the Mann-Whitney test. For all groups, the torque values obtained at the end of 3 months were higher than the torque values obtained at the end of 1 month (Table 6).

**Table 6:** Statistical analysis of 1- and 3-month removal torque test data (Newton/cm)

		n	Mean	Min.	Max.	SD	Mann Whitney testi
Al_2_O_3_	1 month	8	46.11	25.76	80.89	17.4	0.0027
	3 month	8	109.06	45.67	139.16	33.36	
TiO_2_	1 month	8	37.95	20.58	65.47	14.10	0.0005
	3 month	8	93.79	66.15	114.07	14.03	
SiO_2_	1 month	8	40.27	21.26	81.56	20.93	0.0027
	3 month	8	83.19	48.15	104.51	21.45	
Control	1 month	8	42.75	20.93	77.51	17.03	0.0023
	3 month	8	82.06	59.4	124.53	21.79	

The Spearman test was used to determine whether there was a linear relationship between the ISQ values and the torque values. The results from this test showed that there was no linear relationship between the ISQ values and the torque values for all implant groups (Table 7).

**Table 7:** Statistical analysis of the correlation between the final ISQ and torque values (Newton/cm)

								Spearmen test
			n	Mean	Min.	Max.	SD	p
Al_2_O_3_	1 Month	ISQ	8	58.62	51	68	5.39	0.933
		Torque	8	46.11	25.76	80.89	17.4	
	3 Month	ISQ	8	62.75	56	74	6.43	0.61
		Torque	8	109.06	45.67	139.16	33.36	
TiO_2_	1 Month	ISQ	8	54.37	45	69	7.44	0.799
		Torque	8	37.95	20.58	65.47	14.10	
	3 Month	ISQ	8	57.87	49	71	7.18	0.629
		Torque	8	93.79	66.15	114.07	14.03	
SiO_2_	1 Month	ISQ	8	53.87	39	61	6.79	0.456
		Torque	8	40.27	21.26	81.56	20.93	
	3 Month	ISQ	8	59.62	53	63	3.37	0.346
		Torque	8	83.19	48.15	104.51	21.45	
Control	1 Month	ISQ	8	53.62	14	66	16.91	0.713
		Torque	8	42.75	20.93	77.51	17.03	
	3 month	ISQ	8	58.5	48	64	5.04	0.385
		Torque	8	82.06	59.4	124.53	21.79	

## Discussion

There are many methods available for performing the surface modification of dental implants. Foremost among them are bioactive coating applications, chemical applications, and abrasive blasting of the outer layer [10].

Sandblasting is a basic, simple method commonly used for the surface preparation of dental implants. [11] It accelerates osteoblast attachment, and thereby increases osseointegration [12, 13]. If the sand particles are not completely removed from the implant body, they may cause inflammation. In the study, there were no signs of any inflammation and infection.

In the present sandblasting methods, a small negative charge is formed on the titanium. With this feature added to the surface, osseointegration increases [14]. In a study conducted by Guo et al., titanium plates applied to different groups were sandblasted with Al_2_O_3_, and then the static voltage was measured. The results showed the presence of negative static voltage. However, this static voltage decreased over a period of time before becoming stabilized. The amount of this static voltage was reported to be related to environmental factors, such as sandblasting time, sand type, and humidity [14]. This suggests that changing even a small parameter can result in very different surfaces, a fact that should not be overlooked in the creation and development of the surface [15, 16].

Sandblasting not only changes the structure of the surface but also changes the chemistry of the surface and increases the wettability and the potential for early interaction of the implant surface with biological fluids [17, 18].

The most preferred material for dental implants is Al_2_O_3_. Regular roughness values are obtained with particles of different sizes, causing the osteoblast behavior to change and bind to the bone [19, 20]. Al_2_O_3_ has also been reported to stimulate the flow of calcium from the bone [21].

Other particles used as an alternative to Al_2_O_3_ include TiO_2_, hydroxyapatite powders and silicate glass [22–25]. The efficacy of TiO_2_ has been assessed as an alternative to Al_2_O_3_ in many studies. For example, in a study involving implants in dogs, it was observed that surfaces sandblasted with TiO_2_ had more anchoring than the machined surface, although there was no difference in bone-implant contact percentage [26]. This means that histological findings and mechanical test results are not always parallel.

Choi et al. prepared titanium plates that were sandblasted either with bioactive glass particles or with SiO_2_, as well as unsanded plates. The highest roughness ratio was observed in the SiO_2_-blasted plates [27]. A roughened titanium surface enhances the adhesion of protein and the differentiation of bone cells [9, 25, 28, 29].

In the present study, the 1-month and 3-month bone healing in the implants sandblasted with Al_2_O_3_, TiO_2_ and SiO_2,_ and non-blasted implants (control) were investigated. There were no statistically significant differences between the groups in primer stabilization measurements. The similarity of the ISQ values may have been due to the design and geometry being the same for the implants used. There were no statistically significant differences between the ISQ values of the implant groups, and the ISQ was found to increase in the 1-month results. However, the final ISQ average value was higher in the group sandblasted with Al_2_O_3_ implants.

In the comparison of the 1-month and 3-month removal torque values, the 3-month removal torque values were higher in all groups. This result suggests that osseointegration increased over time, regardless of the character of the implant surface.

Ramp and Jeffcoat [30] measured implant stability with RFA in an animal model and found that implant stability and bone support correlated well with histological measurements. In the present study, there was no correlation between the ISQ values and the removal torque values.

During the course of the study conducted by Nedir et al., two implants, one with an ISQ value of 43 and the other with an ISQ value of 46, were lost. Implants determined to have a high ISQ value in Nedir’s study were reported to have no problems. As a result, implants with ISQ values equal to or higher than 47 were considered stable [31]. According to the values prescribed by Osstell Mentor, implants with an ISQ value of over 65 should be considered successful. Studies have shown that implants with ISQ values below 50 have a high failure rate.

Becker et al. conducted a study where 7 of the 100 implants applied on 76 patients failed. The mean initial ISQ value of all the implants applied in the study was 72,1 [32]. Despite having a mean final ISQ value of 66,6, 7 implants still failed. In the present study, 64 implants were used, and the mean initial ISQ value, measured after the operation, was found to be 55,5. Although there were 6 implants with an ISQ value below 50 in the 1st month, primer stabilization was achieved in all implants, and all implants were osseointegrated at the end of both the 1st and the 3rd months, with no losses.

It has been indicated that ISQ values may change due to bone quality. Approximately 2-3 mm of the implants used in this study were in type I bone, with the remaining 7-8 mm in type IV bone. Consequently, the resulting ISQ values may be lower than the values obtained in other studies. Higher ISQ values could be obtained if the iliac bone of the sheep were used. İliac bone can be used in future studies.

Some experimental studies have reported a correlation between the findings from histological and histomorphometric studies – which are accepted as the gold standard – and RFA findings, while others have stated there to be no correlation. Gehrke et al. noted in their study that implants blasted with titanium dioxide particles or aluminum dioxide particles had a good anchorage, with no difference in bone–implant contact [33].

Removal torque test disrupts the bone-implant contact and does not allow for histological examination. Histological studies will require implants which does not applied removal torque. In this study, the number of animals to be used in the study had to be increased. However, given the animal ethics guidelines, this number was to be minimized. For these reasons, histological studies were not performed in our study and this is the limitation of the study.

Ferguson et al. placed 6 different surface implants in the pelvis area of sheep and compared them in 3 phases. The acute phase in the 2nd week, the early phase in the 4th week, and the continuous phase in the 8th week were all assessed using the removal torque test. Six implants were placed in each pelvic iliac bone. The removal torque test values were found to increase over time [34]. In the present study, the comparison of the values in the 1st and 3rd month removal torque showed that the 3-month extraction torque values were higher in all groups.

It is well-known that the removal torque value is influenced by the macro design of the implants. The test and control implants of different surface properties used in this study had the same design and the same groove properties in macroscopic terms. Although there was no statistically significant difference between the removal torque values of the implant groups in the 1st month results of our study, the removal torque value of the Al_2_O_3_ implant group was higher.

## Conclusions

In this study, where four different surfaces were compared, the primer stabilization of all groups was measured using the RFA method, and similar ISQ values were obtained. The similarity of primer stabilization may be because the same design and geometry were used for all the implants. Although there was no significant difference between the ISQ and the removal torque values of the implant groups in the 1-month results, higher values were obtained in the Al_2_O_3_ sandblasted implant group. Statistical binary comparisons yielded higher values on Al_2_O_3_ blasted surfaces, followed by TiO_2_ sandblasted, SiO_2_ sandblasted and non-sandblasted surfaces, although there was no significant difference between removal torque values in the 3-month results. No correlation was found between ISQ values and removal torque values in this study. It was even seen that implants with very low ISQ values had high torque. Overall, the results show that aluminum oxide (Al_2_O_3_) is superior to other sand particles. Future studies to be conducted on this subject should involve larger numbers of animals and implants, as well as histological measurements.

## Abbreviations

Al_2_O_3_: Aluminum oxide
IM: Intramusculary
ISQ: Implant stability quotient
IV: Intravenous
kg: kilogram
mm: millimeter
RFA: Resonance frequency analysis
SiO_2_: Silicon dioxide
TiAlV: Titanium Aluminium Vanadium
TiO_2_: Titanium dioxide
